# Supplementary material for: Rotavirus A Genome Segments Show Distinct Segregation and Codon Usage Patterns
Source: Viruses. 2021 Jul 27;13(8):1460. doi: 10.3390/v13081460 (PMC8402926; doi:10.3390/v13081460)
Supplement: Supplementary file 1 [file viruses-13-01460-s001.zip › Supplementary Figures S1-3.pdf]

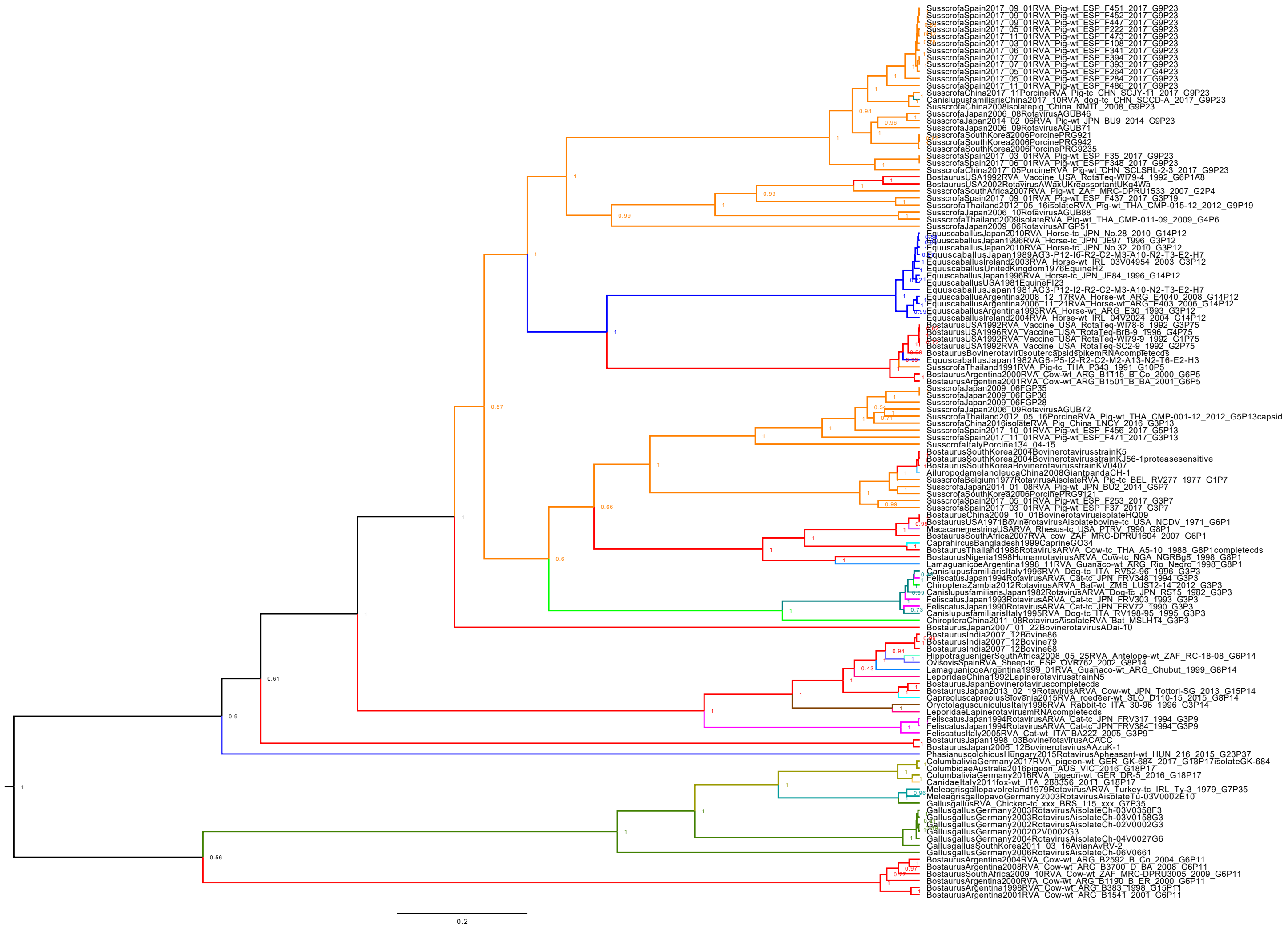

SI Figure S1. Phylogeny of segment 4 used for AI calculation referenced in main text Table 1. The phylogeny was generated using BEAST v1.10.4, based on an alignment of complete segment 9 non-human RVA sequences for which both segment 4 and 9 for each isolate, were available. The branches are colored by host, with the host and strain identifier in the tip label. The tree file is available in SI file 24.

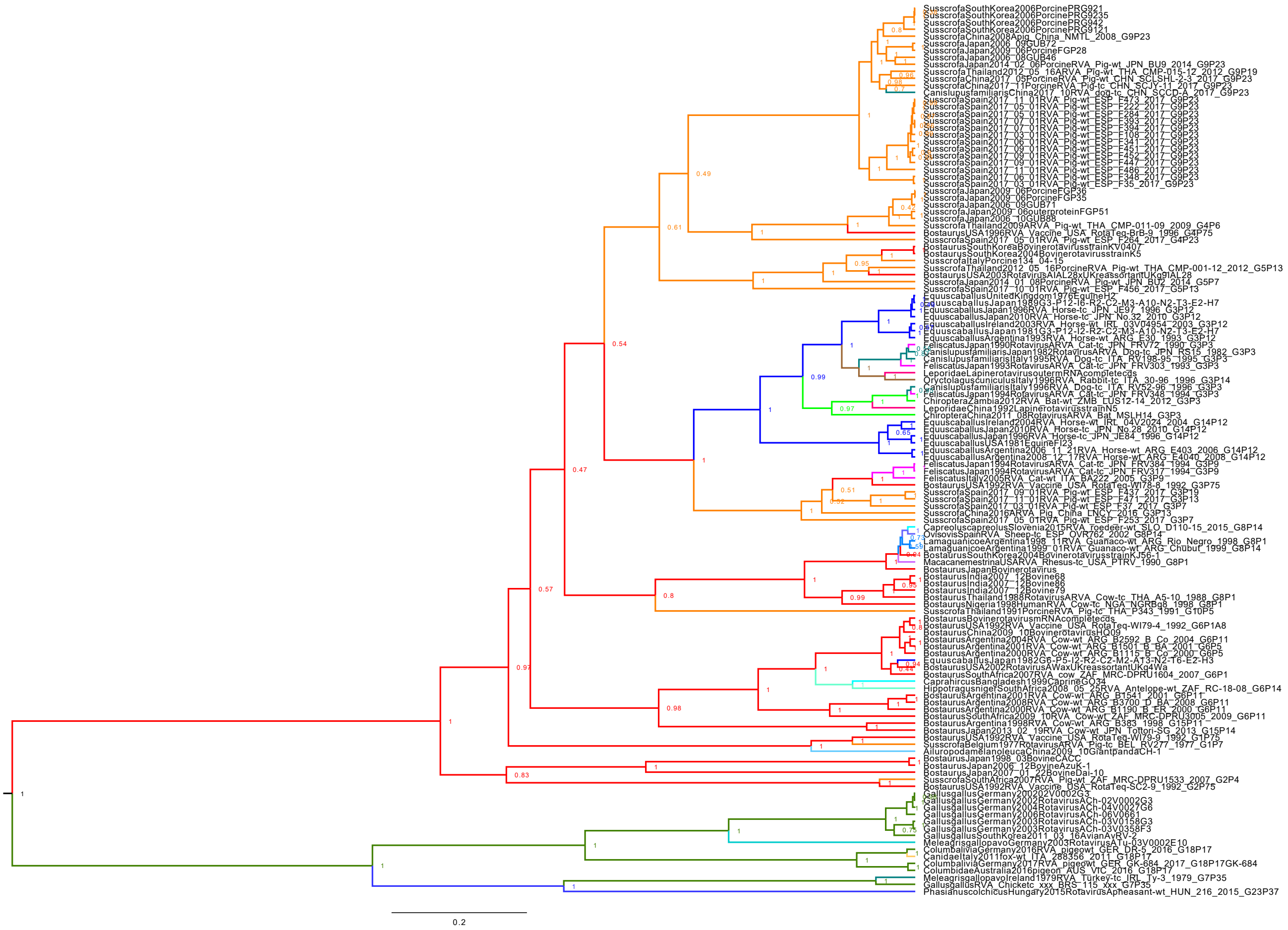

SI Figure S2. Phylogeny of segment 9 used for AI calculation referenced in main text Table 1. The phylogeny was generated using BEAST v1.10.4, based on an alignment of complete segment 9 non-human RVA sequences for which both segment 4 and 9 for each isolate, were available. The branches are colored by host, with the host and strain identifier in the tip label. The tree file is available in SI file 25.

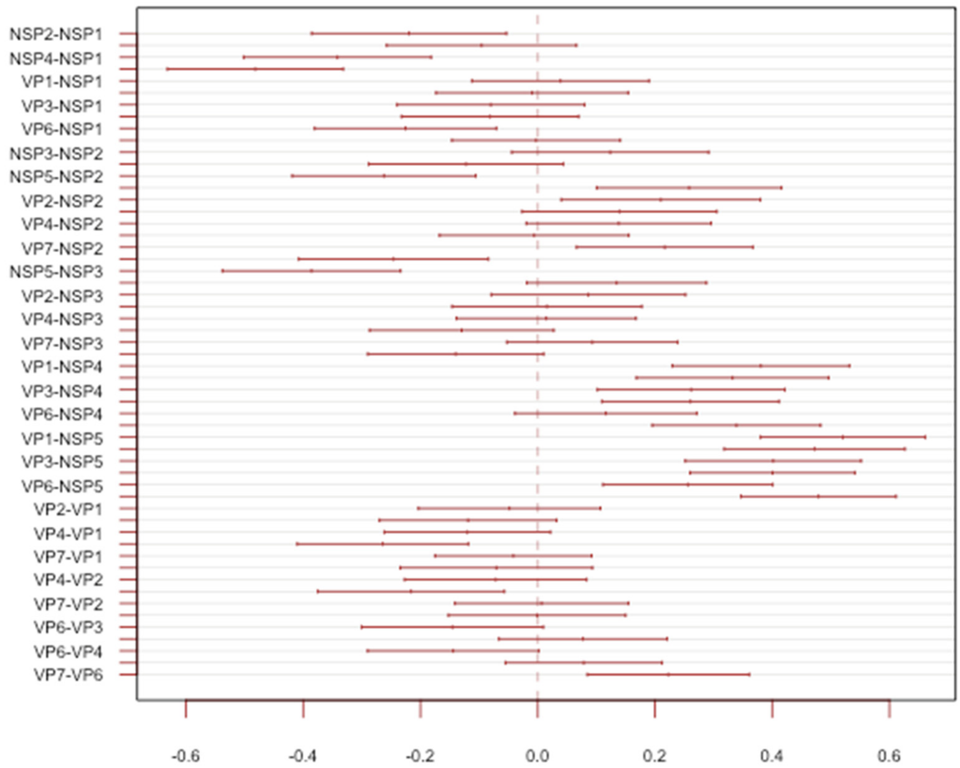

SI Figure S3. Tukey-Kramer 95% pair-wise confidence intervals for main text Figure 7A.
